# Influence of Genetic Polymorphisms on the Short-Term Response to Ranibizumab in Patients With Neovascular Age-Related Macular Degeneration

**DOI:** 10.1167/iovs.64.13.34

**Published:** 2023-10-20

**Authors:** Laura García-Quintanilla, Pablo Almuiña-Varela, Olalla Maroñas, Almudena Gil-Rodriguez, María José Rodríguez-Cid, María Gil-Martinez, Maximino J. Abraldes, Francisco Gómez-Ulla de Irazazabal, Miguel González-Barcia, Cristina Mondelo-Garcia, Raquel Cruz, Ana Estany-Gestal, Maribel Fernández-Rodríguez, Anxo Fernández-Ferreiro

**Affiliations:** 1 Pharmacy Department, University Clinical Hospital of Santiago de Compostela (SERGAS), Santiago de Compostela, Spain; 2 Clinical Pharmacology Group, Health Research Institute of Santiago de Compostela (IDIS), Santiago de Compostela, Spain; 3 Pharmacology, Pharmacy and Pharmaceutical Technology Department, Faculty of Pharmacy, University of Santiago de Compostela (USC), Santiago de Compostela, Spain; 4 Ophthalmology Department, University Clinical Hospital of Santiago de Compostela, (SERGAS), Santiago de Compostela, Spain; 5 Grupo de Genética, Instituto de Investigación Sanitaria de Santiago de Compostela (IDIS), Santiago de Compostela, Galicia, Spain; 6 Grupo de Medicina Xenómica, Centro de Investigación en Medicina Molecular y Enfermedades Crónicas, Universidade de Santiago de Compostela (USC), Santiago de Compostela, Spain; 7 Centro de Investigación Biomédica en Red de Enfermedades Raras, Instituto de Salud Carlos III, Madrid, Spain; 8 Grupo de Medicina Xenómica, Fundación Pública Galega de Medicina Xenómica (FPGMX), Santiago de Compostela, Galicia, Spain; 9 Instituto Oftalmológico Gómez-Ulla, Santiago de Compostela, Spain; 10 Department of Surgery, University of Santiago de Compostela, Santiago de Compostela, Spain; 11 FIDIS-Unidad de Epidemiología e Investigación Clínica, Santiago de Compostela (A Coruña), Spain

**Keywords:** ranibizumab, pharmacogenetics, age-related macular degeneration, anti-vascular endothelial growth factor therapy, treatment response

## Abstract

**Purpose:**

To determine whether genetic risk single nucleotide polymorphisms (SNPs) for age-related macular degeneration (AMD) influence short-term response to intravitreal ranibizumab treatment.

**Methods:**

Forty-four treatment-naive AMD patients were included in a prospective observational study. They underwent three monthly injections of intravitreal ranibizumab for neovascular AMD. After an initial clinical examination (baseline measurement), a follow-up visit was performed to determine treatment response one month after the third injection (treatment evaluation). Patients were evaluated based on ophthalmoscopy, fluorescein angiography, optical coherence tomography (OCT), and OCT angiography. Peripheral venous blood was collected for DNA analysis at baseline visit. Patients were genotyped for single-nucleotide polymorphisms within AMD-relevant genes and classified on good or poor responders based on visual acuity, central retinal thickness, intraretinal fluid, and subretinal fluid.

**Results:**

One hundred ten AMD-associated SNPs have been analyzed. Six were found to be relevant when associated to ranibizumab treatment response. The genetic variants rs890293 (*CYP2J2),* rs11200638 (*HTRA1),* rs405509 *(APOE),* rs9513070 *(FLT1)*, and rs8135665 (*SLC16A8*) predisposed patients to a good response, whereas rs3093077 (CRP) was associated with a poor response. *FTL1*, *SLC16A8*, and *APOE* were the SNPs that showed significance (*P* < 0.05) but did not pass Bonferroni correction.

**Conclusions:**

This is the first study that links novel polymorphisms in genes such as *CRP, SCL16A8*, or *CYP2J2* to treatment response to ranibizumab therapy. On the other hand, *HTRA1, FLT1*, and *APOE* are linked to a good ranibizumab response. These SNPs may be good candidates for short-term treatment response biomarkers in AMD patients. However, further studies will be necessary to confirm our findings.

Age-related macular degeneration (AMD) is the leading cause of irreversible visual impairment among people older than 65 years worldwide.^1^ The neovascular form (nAMD) is characterized by severe vision loss caused by the growth of neo vessels under or within the macula.^2^ Since the approval of anti-vascular endothelial growth factor (anti-VEGF) pharmacotherapy in 2006, the prevalence of legal blindness and visual impairment caused by nAMD has been considerably reduced, removing nAMD from the list of incurable diseases.^3^ The pathophysiology of nAMD is not fully understood; however, many studies have shown it to be multifactorial including genetic factors, metabolic factors, and environmental factors.^4^^,^^5^ The rate of AMD heritability has been estimated at somewhere between 45% to 75%.^6^ The majority of the genes identified for AMD play several parts in susceptibility to disease and pathogenesis,^7^ although it should be noted that the potential association of these genes with treatment response is not as developed.^8^^,^^9^

Ranibizumab, a recombinant fragment of a monoclonal antibody with a high affinity for VEGF-A, was approved for the treatment of nAMD by the United States and the European Union, respectively, in 2006 and 2007 after the phase III clinical trials MARINA and ANCHOR.^10^^–^^13^ Multiple treatment regimens are being used in clinical practice including from monthly dosing, to pro re nata or treat-and-extend strategies.^14^ Regardless of the specific intervention, even though most patients experience a reduction in symptoms after the first intravitreal injection, a pool of patients shows a poor or no response to treatment.^15^^,^^16^ Pharmacogenetics could help us understand the response profiles to treatment by stratifying patients based on their individual genetic profile and optimizing therapeutic decisions to obtain the best efficacy possible and avoid adverse effects of drugs.

PharmGKB, a platform that disseminates knowledge about clinically actionable gene-drug associations,^17^ has clinical annotations on five variants possibly associated with anti-VEGF treatment for AMD. Some of these genetic variants in genes such as *CFB, VEGFA*, and *VEGFR1* suggest an inclination toward a proper response.^9^ On the other hand, variants of *NRP1* and *HTRA1/ARMS* genes have been associated with poor or no response to anti-VEGF treatment,^18^^,^^19^ but the evidence either is substandard or does not support this association because other studies failed to replicate it.^20^^,^^21^ There is a lack of agreement in the pharmacogenetic studies of nAMD response to anti-VEGF treatment, because different periods have been studied such as one, three,^22^^,^^23^ four,^24^ five^25^ or six^26^ months when it comes to short-term response and 12 to 24 months to long-term response.^27^^,^^28^ This makes it difficult for results comparison, particularly, in short-term analysis of the response. Short-term response is the optimal point to measure morphological and functional changes as maximum response is usually achieved at this point^29^^,^^30^ and could help in better clinical management.

Given the high prevalence of AMD and the associated high socioeconomic burden of the anti-VEGF therapies,^31^ it is of the utmost relevance to be able to predict treatment outcome. Therefore a key element for the implementation of individualized therapies is the early identification of poor and non-responders, aiming to maximize visual outcomes while minimizing the number of injections and their associated risks. The purpose of this study is to evaluate the pharmacogenetic relationship between the different genotypes found in the scientific literature of single nucleotide polymorphisms (SNPs) known to be associated with AMD, and response to treatment with ranibizumab for nAMD with the intention to find new SNPs that could predict short-term treatment response.

## Methods and Design

### Characteristics of the Study Group

This was an observational, prospective study of naïve patients with neovascular AMD eligible to start treatment with ranibizumab according to clinical practice. Patients were recruited and evaluated by ophthalmologists from University Clinical Hospital of Santiago de Compostela, Galicia, Spain, from May 2019 to August 2021. After an initial clinical examination (baseline measurement), patients underwent a loading phase that consisted of three-monthly injections with ranibizumab (Lucentis; Novartis, Basel, Switzerland), and a follow-up visit was performed to determine treatment response one month after the third injection (treatment evaluation). Patients were evaluated based on ophthalmoscopy, fluorescein angiography, optical coherence tomography (OCT) (Dri-Triton; Topcon Corp Inc, Tokyo, Japan) and OCT angiography (SS-DRI-Triton-OCTA device; Topcon Corp Inc). This instrument has an A-scan rate of 100,000 scans per second, using a light source with a wavelength of 1 µm for a deeper penetration into tissue. All patients in this study were imaged using the 3 × 3 mm, 6 × 6 mm, and 9 mm scan patterns centered on the fovea. OCT angiography images were processed, and measures were analyzed using IMAGEnet 6.

The study and the data collection strictly adhered to the declaration of Helsinki principles. Ethical approval was given by the local Institutional Review Board and the autonomous region of Galicia (2017/614).

### Patient's Criteria

Patients had to meet all the inclusion criteria, no exclusion criteria and had to give their consent for the collection and storage of blood samples. Inclusion criteria were defined as neovascularization secondary to AMD, age over 55 years, and visual acuity (VA) greater than 25 Early Treatment Diabetic Retinopathy Study (ETDRS) letters at baseline. Exclusion criteria included any retinal disease other than nAMD. In addition, patients who had previously undergone any ophthalmologic surgery other than cataract surgery or had signs of intraocular inflammation were also excluded. None of the subjects had received previous anti-VEGF treatment for nAMD.

### Measures of Response to Treatment

Treatment response analysis to ranibizumab therapy was performed four months after the initiation of ranibizumab treatment. The classification of this response is based on Amoaku et al.^32^ Patients with intraretinal fluid (IRF) or subretinal fluid (SRF) resolution, reduced central retinal thickening (CRT), and improvement of at least five ETDRS letters were considered good responders. Patients with less than 25% decrease from baseline on OCT-CRT, no change in VA (<5 letters), and continued or new IRF, SRF after anti-VEGF therapy were classified as poor or nonresponders.

### Genotype Determination

Peripheral venous blood was collected in two EDTA tubes (4 mL) at the baseline visit. The separation of leukocytes in blood was done within 24 hours. Five milliliters of blood were spun in a centrifuge at 2280*g* for 15 minutes at room temperature. After the leukocytes were separated from the other components of blood, they were washed twice with saline solution, and they were resuspended in 500 µL of saline solution. Afterward, the leukocytes samples were stored at −80°C until the DNA isolation.

DNA was isolated following the manufacturer's instructions with the Wizard Genomic DNA Purification Kit (Promega Corporation, Madison, WI, USA). DNA concentration and purity were measured with Qubit 2.0 (Thermo Fisher Scientific, Waltham, MA, USA) and NanoDrop 1000 Spectrophotometer (Thermo Fisher Scientific). For the panel design, genotyping was performed using a custom made OpenArray technology using the QuantStudio 12K Flex System (Thermo Fisher Scientific), which is a real-time PCR system that uses Taqman technology. The results were analyzed using two software, the QuantStudio 12K Flex Real-Time PCR Software and Taqman Genotyper Software, which allows a cluster analysis by biomarker.

A selection of DNA biomarkers has been carried out through the development of a bibliographic search based on scientific evidence so far on articles related to AMD and pharmacogenetics. Fifty-seven articles between the years 2004 to 2021 have been reviewed.^33^ A total of 110 of AMD-associated single nucleotide polymorphisms have been analyzed (SNP selected in Supplementary Table S1). Biomarkers that emerged as significant in prior research and any biomarkers that leaned toward significance have been selected, despite not being particularly significant in the study. The chosen biomarkers belonged to both treatment response biomarkers and AMD susceptibility and pathogenesis biomarkers.

### Statistical Analysis

Pharmacogenetic data were analyzed with PASW Statistics 20 (IBM SPSS Statistics; IBM, Armonk, NY, USA) and R for Windows. Differences between good responder versus poor responder groups were explored using statistical tests; nonparametric Mann-Whitney test in the case of quantitative variables, and chi-square test or Fisher's exact test in the case of categorical variables. A logistic regression analysis was performed to detect the genetic association for each SNP and response to ranibizumab treatment using codominant, dominant, recessive, and log-additive genetic models, with sex used as covariate. Correction (for multiple tests) was performed via the Bonferroni method.

## Results

### Cohort Description

This was an observational study where 44 naïve patients have been included. The demographic and response parameters of the study population are summarized in Table 1. The median age was 80 years old with 75% of the patients being over the age of 75 at the start of treatment with ranibizumab. 59% of the patients were women with a worse outcome than men in the short-term response to ranibizumab treatment (*P* = 0.014).

**Table 1.** Demographic and Clinical Parameters

	Total (*n* = 44)	Poor Responders (*n* = 20)	Good Responders (*n* = 24)	*P* Value
Sex (women)	26 (59.1%)	16 (80.0%)	10 (41.7%)	**0.014**
Age Median, IQR	80 (74–86)	80 (74–86)	79 (75–86)	**0.850**
BMI (kg/m^2^) Median, IQR	29.0 (25.6–31.2)	27.6 (24.5–31.6)	29.4 (25.9–31.3)	**0.302**
Response parameters				
VA (ETDRS)				
Basal	60 (45–70)	58 (40–71)	60 (50–66)	**0.715**
Treated	66 (55–75)	62 (51–73)	70 (65–75)	**0.029**
CRT (µm)				
Basal	298 (260–354)	328 (273–395)	292 (251–343)	**0.229**
Treated	214 (194–250)	218 (198–274)	212 (190–242)	**0.328**
IRF (%)				
Basal	27 (61.4%)	11 (55.0%)	16 (66.7%)	**0.539**
Treated	8 (18.2%)	5 (25.0%)	3 (12.5%)	**0.436**
SRF				
Basal	33 (75.0%)	17 (85.0%)	16 (66.7%)	**0.169**
Treated	9 (20.5%)	8 (40.0%)	1 (4.2%)	**0.006**

BMI, body mass index.

Regarding the analysis of the response at the end of the loading phase, 52.3% of our series presented a complete response to treatment (gain ≥5 ETDRS letters and disappearance of fluid), 43.2% presented a partial response (gain <5 ETDRS letters or persistence of any type of fluid), and 4.5% did not present any type of response. Nonresponders and poor responders were pooled in a single group for analysis purposes.

The median visual acuity was 60 ETDRS letters (interquartile range [IQR], 45–70) and the median CRT was 298 µ (IQR, 260–354) at the initial visit. In the OCT parameters: 61.7% of the eyes had IRF at diagnosis, 75% SRF. None of the patients presented areas of geographic atrophy as defined by the Classification of Atrophy Meetings at baseline.^34^

**Figure 1.**
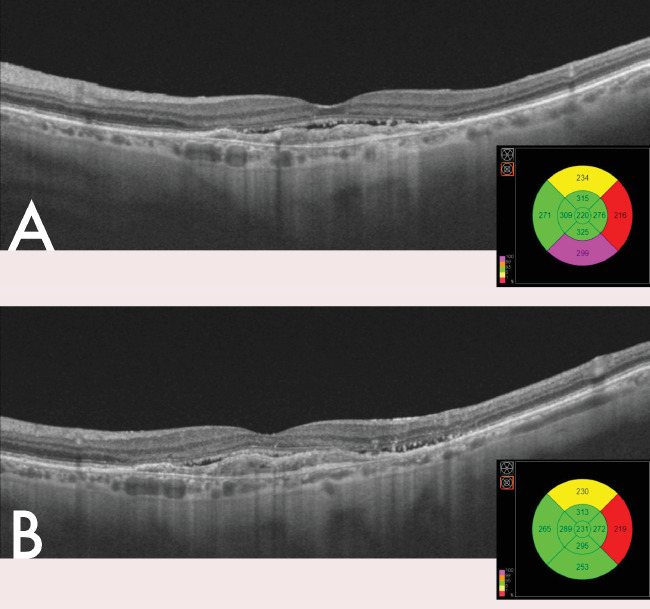
Macular neovascularization (MNV) without response to treatment. (**A**) Baseline image SRF can be observed over MNV. (**B**) Image after loading phase were SRF can be seen, along with an increase in the central subfield of the thickness of the ETDRS grid.

**Figure 2.**
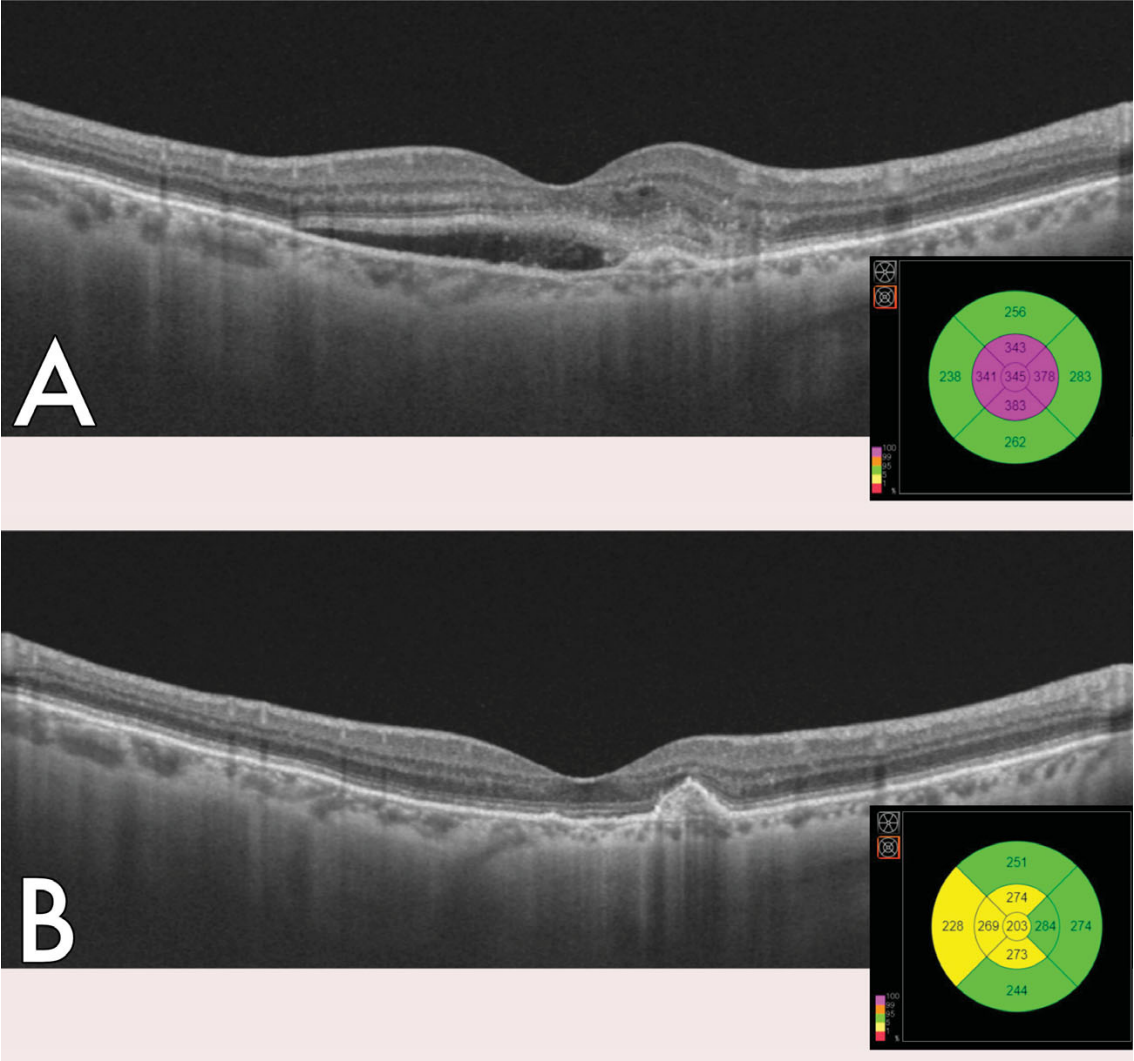
Macular neovascularization (MNV) showing response to ranibizumab treatment. (**A**) Baseline image were IRF and SRF can be observed over MNV. (**B**) Image after loading phase without any type of fluid have can be seen, along with a recuperation of the normal thickness in all of the subfields of the ETDRS grid.

At the first visit after completing the loading phase, the median VA was 66 ETDRS letters (IQR, 55–75) and the median CRT was 214 µm (IQR, 194–250). We found IRF in just 18.1 % of the cases, and SRF in 20.5%. No intraretinal hemorrhages were found in any of our patients. Regarding the improvement of VA, an overall improvement of 7 ± 13 letters has been quantified after the loading phase (Figs. 1, 2).

Good responders did not differ in VA from poor responders at baseline (*P* = 0.715), but we found significant differences in VA between groups after the loading phase (*P* = 0.029). These differences were not found between responders and poor responders in the baseline CRT, nor after treatment.

Median CRT decreased significantly over time (*P* < 0.0001) in both responders (292 µm; IQR, 251–343 to 212 µm; IQR, 190–242) and poor responders (328 µm; IQR, 273–395 to 218 µm; IQR, 198–274). VA surged over time in both groups, but it was found to have statistical significance only in the responders’ group (*P* < 0.0001).

### Genetic Effectiveness Outcomes

A total of 110 of AMD-associated single nucleotide polymorphisms have been analyzed (Supplementary Table S1). No polymorphism was excluded because of noncompliance with the established quality control. All polymorphisms fulfilled the Hardy-Weinberg equilibrium. The genetic association between the selected SNPs with the different types of treatment response was explored (Supplementary Table S2). The SNPs with a significant association in the logistic regression analysis are shown in Table 2. The odds ratio (OR) reports the effect of the rare allele in the study (the frequent homozygote is always taken as reference). The rest of the SNP evaluated in this study showed no association with ranibizumab treatment (Supplementary Table S2).

**Table 2.** SNPs That Showed Significant Association With Response in Any of the Tested Models

Gen	SNP	Genotype	Good Responder	Poor Responder	cOR (95% CI)	*P* Value	aOR (95% CI)	*P* Value
*SLC16A8*	rs8135665	CC	9 (37.5%)	13 (68.4%)	0.28 (0.08–0.99)	0.042^*^	0.17 (0.04–0.79)	0.016^*^
		CT+TT	15 (62.5%)	6 (31.6%)				
*CYP2J2*	rs890293	CC	24 (100%)	15 (78.9%)	—	0.031^*^	—	0.084
		AC	0 (0%)	4 (21.1%)				
*FLT1*	rs9513070	AA+AG	23 (95.8%)	14 (73.7%)	8.21 (0.87–77.74)	0.072	17.35 (1.3–231.33)	0.010^*^
		GG	1 (4.2%)	5 (26.3%				
*APOE*	rs405509	TT	5 (20.8%)	9 (47.4%)	0.29 (0.08–1.11)	0.065	0.15 (0.03–0.84)	0.016^*^
		GT+GG	19 (79.2)	10 (52.6%)				
*HTRA1*	rs11200638	GG	5 (21.7%)	9 (52.9%)	0.25 (0.06–0.98)	0.040^*^	0.32 (0.07–1.44)	0.131
		AG+AA	18 (78.3%)	8 (47.1%)				
*CRP*	rs3093077	AA	23 (95.8%)	13 (68.4%)	10.62 (1.15–98.09)	0.032^*^	6 (0.6–60.44)	0.085
		AC	1 (4.2%)	6 (31.6%)				

cOR, crude odds ratio; aOR, adjusted odds ratio by sex.

* These SNPs did not reach significance after the Bonferroni correction.

Six SNPs were found to be relevant in their association to treatment response with ranibizumab. The SNPs that had better response to ranibizumab treatment were rs890293 of *CYP2J2* (cytochrome P450 2J2) gene, rs11200638 of *HTRA1* (human high temperature requirement serine protease A1) and rs8135665 of *SLC16A8* (solute carrier family 16 member 8) gene. On the other hand, risk alleles of rs3093077 of C-reactive protein (CRP) and rs9513070 of FTL1 suggested worse response. However, these SNPs did not reach significance after the Bonferroni correction.

The allele T (minority allele) of rs8135665 (*SLC16A8*) was associated with a risk of better response to treatment with ranibizumab after the loading dose in a dominant model. This SNP not only preserved significance, but it also increased once the model was adjusted for sex.

No homozygotes for the rare allele (A) of rs890293 (*CYP2J2*) were found, and heterozygotes only appeared in the group of non-responders, so the C allele may be beneficial when patients were treated with ranibizumab. The allele G of rs405509 (*APOE*) was associated with improved ranibizumab treatment outcomes after the loading dose in a dominant model when the model was adjusted for sex. In the case of rs9513070 of *FLT1* gene, the G allele is the risk allele and tends to be frequent in patients with a substandard response to treatment with ranibizumab therapy in a recessive model.

The SNP that appeared to have worse response was rs3093077 of *CRP* gene where the C allele had higher frequency of heterozygotes in poor responders, but this SNP failed to display any association once the analysis was corrected for sex (*P* = 0.0856). It should be noted that the count was excessively low in the response group of this SNP.

The frequent allele (G) of rs11200638; *HTRA1*, had the worst response to treatment because it appeared more frequently in the group of nonresponders (*P* = 0.0404; OR = 0.25; 95% CI, 0.06–0.98), but it also did not signify any association once the analysis was corrected for sex (*P* = 0.1318).

## Discussion

The main purpose of pharmacogenetics is the possibility of stratifying patients based on their individual genetic profile and to provide them with the most appropriate therapeutic decision to avoid lack of efficacy and adverse effects of drugs. Therefore early identification of nonresponders or poor responders is essential for the implementation of personalized therapies in the hope of maximizing visual outcomes and minimizing the number of treatments and associated risks.

We have analyzed biomarkers related both to response and to the development of macular neovascularization. Although the former is more scarce than the latter in the literature, both provide new insights of treatment response.

The *SLC16A8* gene encodes a monocarboxylate transporter protein (MCT3) that is specifically expressed by the RPE and is involved in the transport of pyruvate, with lactate and ketones^35^ mediating the level of acidity in the outer retinal segments and preventing the accumulation of lactate in the subretinal space. The rs8135665 variant is of interest because it is predicted to disrupt the processing of the encoded transcript^36^ leading to defective lactate transport that may ultimately cause retinal acidification and photoreceptor dysfunction. Interestingly, *SLC16A8* gene knock-out mice have changes in visual function,^37^ but the overall retinal histology remains normal.

*SLC16A8* has been linked to susceptibility to AMD,^36^^,^^38^^,^^39^ but no data have been found in the literature linking it to response to treatment. Ours is the first study to find an association between SLC16A8 and response to ranibizumab treatment, and the prevalence and trend of the data shown allows us to establish the importance of increasing the sample size to establish conclusively whether it is a protective factor of good response.

*CYP2J2* belongs to the family of cytochromes that are common in the heart and vascular endothelium.^40^ There is little published on the role of *CYP2J2* in nAMD. It has been observed that products from *CYP2J2* metabolism, induced angiogenesis in vivo and ex vivo in mice, and inhibition of *CYP2J2* may suppress pathological vessel growth in choroidal neovascularization.^41^ Liutkeviciene et al. aimed to determine the relationship between early neovascular AMD and the rs890293 polymorphism of the *CYP2J2* gene in a Lithuanian population. The *CYP2J2* TT genotype was statistically less frequent in patients >65 years with AMD than in controls, suggesting that polymorphism rs890293 TT could be a protective genotype.^42^

In our case, we found no homozygotes for the rare allele (A) and heterozygotes were only found in the group of non-responders, so the C allele could be beneficial when patients are treated with ranibizumab. However, it is true that the frequency of the TT genotype is very low according to the scientific literature and it is similar to that obtained in our population (4.7%). This rarity has also been observed in other pathologies such as Alzheimer's disease^40^ and atherosclerosis.^43^ Ours is also the first study to find association between *CYP2J2* and response to ranibizumab treatment.

*FLT1* is a gene which encodes a member of the vascular endothelial growth factor receptor (VEGFR) family. This protein binds to VEGFR-A, VEGFR-B, and placental growth factor and plays an important role in angiogenesis.^44^ There are several studies that relate different SNPs of this gene (rs7993418, rs9582036) with improved response in AMD.^9^^,^^44^ In our study, these SNPs did not reach significance. The rs9513070 is the variant that holds significance in our case, where the G allele is the risk allele and is more common in patients with a substandard response to treatment with ranibizumab. However, the small sample size does not allow for a perfect interpretation of risk quantification because of the width of the confidence interval (OR = 17.35; 95% CI, 1.3-231.33). Nonetheless, these results are consistent with a retrospective study of a Belgian population treated with anti-VEGF where the A allele was also associated with improved response to short-term treatment (as in our case), but it failed to maintain association when evaluated again a year later.^44^

The APOE polymorphic protein acts by revamping the homeostasis of cholesterol and other lipids. It is the major apolipoprotein of the central nervous system, and it has been shown to be a component of drusen, and can accumulate in the cytoplasm of RPE cells.^45^ The rs405509 variant that was significant in our study was previously related to AMD risk, but it did not identify additional risks than the other SNPs related to this gene.^46^ No other studies have been discovered in relation to AMD treatment response regarding this SNP. Alternatively, two SNPs, rs7412 and rs4229358, which did not reach statistical significance in our study, were found to confer significantly better visual outcomes after anti-VEGF at short-term response.^47^^,^^48^

Genetic variants of *CRP* have been shown to determine serum levels of this protein, particularly in response to inflammatory stimuli.^49^ Several SNPs of *CRP* gene have been studied in relation to treatment response but related to photodynamic therapy (PDT).^50^ Two variants related to this gene, rs2808635 and rs876538, did find an association between with response to PDT, but rs3093077 was not significative. No other studies have been found that relates to anti-VEGF treatment, being this the first one that explores this possible association with the rs3093077 variant.

Finally, the *HTRA1* gene encodes a heat shock serine protease that regulates TGF-β.^51^ The *HTRA1* gene is one of the few genes that have been linked to a poor response to anti-VEGF treatment and is included in PharmGKB database,^17^ although still with a very low level of evidence (level 4) or association. Some studies show that the AA risk genotype is more frequent among nonresponders or poor responders.^22^^,^^52^ However, in our case, the AA allele of rs11200638 is protective, with the most frequent allele (GG) having the worst response to treatment because it appears more frequently in the group of nonresponders. Our results are consistent with two studies, one with short-term response^19^ to ranibizumab treatment, and another in long-term response to bevacizumab.^53^ Also, homozygotes (GG or AA) seem to have poorer response than individuals who were heterozygous for the risk A allele heterozygotes (GA).^54^ We found no relation to other biomarkers known to affect treatment response such as *VEGF*, *CFH* or *ARMS* as detailed in PharmGKB probably because of the small sample size that was recruited for this study.

The primary limitation of this study is undoubtedly the sample size. However, this limitation originates from the strict inclusion and exclusion criteria, along with the use of a sole center for recruitment. Likewise, this work only shows the early response associated to loading phase. Short-term response was chosen because previous studies revealed that the optimal point to measure morphological and functional response is following the loading phase where maximum response is seen.^11^^,^^29^ Analyzing short-term response allows for an improved understanding of the visual potential, helping to manage patients' expectations and providing guidance on clinical management.^32^ On the other hand, with long-term follow-up and an individualized injection schedule, the genetic profile associated with the visual outcome may change, as has been seen in other studies.^9^^,^^25^^,^^55^^,^^44^

Overall, we have only analyzed the response to treatment of nAMD with ranibizumab as anti-VEGF agent as our goal was to be able to identify the patients who could benefit the most from this treatment. This grants an opportunity to further expand this area of research as other approved agents for nAMD also exert their effect in the VEGF pathway, such as brolucizumab, aflibercept or faricimab. Surveying the genetic profiles associated to response to varying agents could offer the opportunity to customize the treatment of patients. On the other hand, due to the multifactorial pathophysiology of the disease, the treatment response may be further be explored with studies that bridge the gap between genetic markers and clinical imaging biomarkers^56^^,^^57^ more readily available to daily practice such as choroidal features such as choroidal thickness and perfusion index^58^ or new OCT angiography biomarkers such as three-dimensional characterization^59^ that might help in future clinical decisions.

## Conclusions

Our study provides further insight into the pharmacogenetics of the clinical response of nAMD to ranibizumab therapy, by identifying novel polymorphism in genes such as *SCL16A8* or *CYP2J2* that are associated with better outcome to treatment response. This is the first study that links *CRP* genotype, which previously was only studied in PDT, to anti-VEGF therapy obtaining a good response in homozygotes. *FTL1* confirms prior published data regarding positive response, on the other hand, in this study *HTRA1* risk allele was associated with a good ranibizumab response. Based on our results, *SCL16A8*, *CYP2J2*, *FLT1,* *APOE*, and *CRP* may be good candidates for treatment response biomarkers in AMD patients. However, further studies will be necessary to confirm our findings.

## Supplementary Material

Supplement 1

## References

[1] Li JQ, Welchowski T, Schmid M, Mauschitz MM, Holz FG, Finger RP. Prevalence and incidence of age-related macular degeneration in Europe: a systematic review and meta-analysis. *Br J Ophthalmol*. 2020; 104: 1077–1084. 31712255 10.1136/bjophthalmol-2019-314422

[2] Arrigo A, Saladino A, Aragona E, et al. Different outcomes of anti-VEGF treatment for neovascular AMD according to neovascular subtypes and baseline features: 2-year real-life clinical outcomes. *Biomed Res Int*. 2021; 2021: 5516981. 34124243 10.1155/2021/5516981 PMC8169263

[3] Schmidt-Erfurth U, Chong V, Loewenstein A, et al. Guidelines for the management of neovascular age-related macular degeneration by the European Society of Retina Specialists (EURETINA). *Br J Ophthalmol*. 2014; 98: 1144–1167. 25136079 10.1136/bjophthalmol-2014-305702 PMC4145443

[4] Pugazhendhi A, Hubbell M, Jairam P, Ambati B. Neovascular macular degeneration: a review of etiology, risk factors, and recent advances in research and therapy. *Int J Mol Sci*. 2021; 22: 1170. 33504013 10.3390/ijms22031170 PMC7866170

[5] García-Quintanilla L, Rodríguez-Martínez L, Bandín-Vilar E, et al. Recent advances in proteomics-based approaches to studying age-related macular degeneration: a systematic review. *Int J Mol Sci*. 2022; 23: 14759. 36499086 10.3390/ijms232314759 PMC9735888

[6] DeAngelis MM, Owen LA, Morrison MA, et al. Genetics of age-related macular degeneration (AMD). *Hum Mol Genet*. 2017; 26(R1): R45–R50. 28854576 10.1093/hmg/ddx228 PMC5886461

[7] Yan Q, Ding Y, Weeks DE, Chen W. AMD genetics: methods and analyses for association, progression, and prediction. *Adv Exp Med Biol*. 2021; 1256: 191–200. 33848002 10.1007/978-3-030-66014-7_7

[8] Cruz-Gonzalez F, Cabrillo-Estévez L, López-Valverde G, Cieza-Borrella C, Hernández-Galilea E, González-Sarmiento R. Predictive value of VEGF A and VEGFR2 polymorphisms in the response to intravitreal ranibizumab treatment for wet AMD. *Graefes Arch Clin Exp Ophthalmol*. 2014; 252: 469–475. 24522370 10.1007/s00417-014-2585-7

[9] Cobos E, Recalde S, Anter J, et al. Association between CFH, CFB, ARMS2, SERPINF1, VEGFR1 and VEGF polymorphisms and anatomical and functional response to ranibizumab treatment in neovascular age-related macular degeneration. *Acta Ophthalmol*. 2018; 96(2): e201–e212. 28926193 10.1111/aos.13519

[10] Rosenfeld PJ, Brown DM, Heier JS, et al. Ranibizumab for neovascular age-related macular degeneration. *N Engl J Med*. 2006; 355: 1419–1431. 17021318 10.1056/NEJMoa054481

[11] Brown DM, Michels M, Kaiser PK, et al. Ranibizumab versus verteporfin photodynamic therapy for neovascular age-related macular degeneration: two-year results of the ANCHOR study. *Ophthalmology*. 2009; 116: 57–65.e5. 19118696 10.1016/j.ophtha.2008.10.018

[12] Rofagha S, Bhisitkul RB, Boyer DS, Sadda SR, Zhang K, SEVEN-UP Study Group. Seven-year outcomes in ranibizumab-treated patients in ANCHOR, MARINA, and HORIZON: a multicenter cohort study (SEVEN-UP). *Ophthalmology*. 2013; 120: 2292–2299. 23642856 10.1016/j.ophtha.2013.03.046

[13] Bressler NM, Chang TS, Suñer IJ, et al. Vision-related function after ranibizumab treatment by better- or worse-seeing eye: clinical trial results from MARINA and ANCHOR. *Ophthalmology*. 2010; 117: 747–756.e4. 20189654 10.1016/j.ophtha.2009.09.002

[14] Gil-Martínez M, Santos-Ramos P, Fernández-Rodríguez M, et al. Pharmacological advances in the treatment of age-related macular degeneration. *Curr Med Chem*. 2020; 27: 583–598. 31362645 10.2174/0929867326666190726121711

[15] Chaudhary V, Matonti F, Zarranz-Ventura J, Stewart MW. Impact of fluid compartments on functional outcomes for patients with neovascular age-related macular degeneration: a systematic literature review. *Retina*. 2022; 42: 589–606. 34393212 10.1097/IAE.0000000000003283 PMC8946587

[16] Shuang YG, Kim BJ, Maguire MG, et al. Sustained visual acuity loss in the comparison of age-related macular degeneration treatments trials. *JAMA Ophthalmol*. 2014; 132: 915–921. 24875610 10.1001/jamaophthalmol.2014.1019 PMC4151260

[17] Thorn CF, Klein TE, Altman RB. PharmGKB: the pharmacogenomics knowledge base. *Methods Mol Biol*. 2013; 1015: 311–320. 23824865 10.1007/978-1-62703-435-7_20 PMC4084821

[18] Lorés-Motta L, de Jong EK, den Hollander AI. Exploring the use of molecular biomarkers for precision medicine in age-related macular degeneration. *Mol Diagn Ther*. 2018; 22: 315–343. 29700787 10.1007/s40291-018-0332-1 PMC5954014

[19] Mohamad NA, Ramachandran V, Mohd Isa H, et al. Association of HTRA1 and ARMS2 gene polymorphisms with response to intravitreal ranibizumab among neovascular age-related macular degenerative subjects. *Hum Genomics*. 2019; 13: 13. 30795802 10.1186/s40246-019-0197-3 PMC6387522

[20] Hagstrom SA, Ying GS, Pauer GJT, et al. Pharmacogenetics for genes associated with age-related macular degeneration in the Comparison of AMD Treatments Trials (CATT). *Ophthalmology*. 2013; 120: 593–599. 23337555 10.1016/j.ophtha.2012.11.037 PMC3633658

[21] Lotery AJ, Gibson J, Cree AJ, et al. Pharmacogenetic associations with vascular endothelial growth factor inhibition in participants with neovascular age-related macular degeneration in the IVAN Study. *Ophthalmology*. 2013; 120: 2637–2643. 24070809 10.1016/j.ophtha.2013.07.046

[22] Imai D, Mori K, Horie-Inoue K, et al. CFH, VEGF, and PEDF genotypes and the response to intravitreous injection of bevacizumab for the treatment of age-related macular degeneration. *J Ocul Biol Dis Infor*. 2010; 3: 53–59. 21811649 10.1007/s12177-010-9055-1 PMC3148139

[23] Smailhodzic D, Muether PS, Chen J, et al. Cumulative effect of risk alleles in CFH, ARMS2, and VEGFA on the response to ranibizumab treatment in age-related macular degeneration. *Ophthalmology*. 2012; 119: 2304–2311. 22840423 10.1016/j.ophtha.2012.05.040

[24] Strunz T, Pöllmann M, Gamulescu MA, Tamm S, Weber BHF. Genetic association analysis of anti-VEGF treatment response in neovascular age-related macular degeneration. *Int J Mol Sci*. 2022; 23: 6094. 35682771 10.3390/ijms23116094 PMC9181567

[25] Park UC, Shin JY, Kim SJ, et al. Genetic factors associated with response to intravitreal ranibizumab in Korean patients with neovascular age-related macular degeneration. *Retina*. 2014; 34: 288–297. 23842101 10.1097/IAE.0b013e3182979e1e

[26] Chang W, Noh DH, Sagong M, Kim IT. Pharmacogenetic association with early response to intravitreal ranibizumab for age-related macular degeneration in a Korean population. *Mol Vis*. 2013; 19: 702–709. 23559864 PMC3611944

[27] Miyake M, Yamashiro K, Akagi-Kurashige Y, et al. Vascular endothelial growth factor gene and the response to anti-vascular endothelial growth factor treatment for choroidal neovascularization in high myopia. *Ophthalmology*. 2014; 121: 225–233. 23953100 10.1016/j.ophtha.2013.06.043

[28] Lazzeri S, Orlandi P, Piaggi P, et al. IL-8 and VEGFR-2 polymorphisms modulate long-term functional response to intravitreal ranibizumab in exudative age-related macular degeneration. *Pharmacogenomics*. 2016; 17: 35–39. 26653034 10.2217/pgs.15.153

[29] Heier JS, Brown DM, Chong V, et al. Intravitreal aflibercept (VEGF trap-eye) in wet age-related macular degeneration. *Ophthalmology*. 2012; 119: 2537–2548. 23084240 10.1016/j.ophtha.2012.09.006

[30] Gale RP, Finger RP, Eldem B, et al. The management of neovascular age-related macular degeneration: a systematic literature review of patient-reported outcomes, patient mental health and caregiver burden. *Acta Ophthalmol*. 2023; 101(1): e26–e42. 35790079 10.1111/aos.15201 PMC10084380

[31] Moisseiev E, Tsai YL, Herzenstein M. Treatment of neovascular age-related macular degeneration: an economic cost-risk analysis of anti-vascular endothelial growth factor agents. *Ophthalmol Retina*. 2022; 6: 205–212. 34454123 10.1016/j.oret.2021.08.009

[32] Amoaku WM, Chakravarthy U, Gale R, et al. Defining response to anti-VEGF therapies in neovascular AMD. *Eye (Lond)*. 2015; 29: 1397–1398. 26446737 10.1038/eye.2015.159 PMC5176328

[33] Maroñas O, García-Quintanilla L, Luaces-Rodríguez A, et al. Anti-VEGF treatment and response in age-related macular degeneration: disease's susceptibility, pharmacogenetics and pharmacokinetics. *Curr Med Chem*. 2020; 27: 549–569. 31296152 10.2174/0929867326666190711105325

[34] Sadda SR, Guymer R, Holz FG, et al. Consensus definition for atrophy associated with age-related macular degeneration on OCT: classification of atrophy report 3. *Ophthalmology*. 2018; 125: 537–548. 29103793 10.1016/j.ophtha.2017.09.028 PMC11366072

[35] Halestrap AP. The SLC16 gene family - structure, role and regulation in health and disease. *Mol Aspects Med*. 2013; 34(2–3): 337–349. 23506875 10.1016/j.mam.2012.05.003

[36] Fritsche LG, Igl W, Bailey JNC, et al. A large genome-wide association study of age-related macular degeneration highlights contributions of rare and common variants. *Nat Genet*. 2016; 48: 134–143. 26691988 10.1038/ng.3448 PMC4745342

[37] Daniele LL, Sauer B, Gallagher SM, Pugh EN, Philp NJ. Altered visual function in monocarboxylate transporter 3 (Slc16a8) knockout mice. *Am J Physiol Cell Physiol*. 2008; 295(2): C451–457. 18524945 10.1152/ajpcell.00124.2008 PMC2518420

[38] Cascella R, Strafella C, Caputo V, et al. Towards the application of precision medicine in age-related macular degeneration. *Prog Retin Eye Res*. 2018; 63: 132–146. 29197628 10.1016/j.preteyeres.2017.11.004

[39] Fritsche LG, Chen W, Schu M, et al. Seven new loci associated with age-related macular degeneration. *Nat Genet*. 2013; 45: 433–439. 23455636 10.1038/ng.2578 PMC3739472

[40] Yan H, Kong Y, He B, et al. CYP2J2 rs890293 polymorphism is associated with susceptibility to Alzheimer's disease in the Chinese Han population. *Neurosci Lett*. 2015; 593: 56–60. 25796175 10.1016/j.neulet.2015.03.024

[41] Gong Y, Tomita Y, Edin ML, et al. Cytochrome P450 oxidase 2J inhibition suppresses choroidal neovascularization in mice. *Metabolism*. 2022; 134: 155266. 35868524 10.1016/j.metabol.2022.155266 PMC9535696

[42] Liutkeviciene R, Vilkeviciute A, Botov R, Botova O, Buteikiene D, Kriauciuniene L. Associations between CYP2J2 (-76G>T) rs890293 polymorphism and age-related macular degeneration. *Biomed Pap Med Fac Univ Palacky Olomouc Czech Repub*. 2020; 164: 267–272. 31132075 10.5507/bp.2019.019

[43] Lee CR, North KE, Bray MS, Couper DJ, Heiss G, Zeldin DC. CYP2J2 and CYP2C8 polymorphisms and coronary heart disease risk: the Atherosclerosis Risk in Communities (ARIC) study. *Pharmacogenet Genomics*. 2007; 17: 349–358. 17429317 10.1097/FPC.0b013e32809913ea PMC1947003

[44] Balikova I, Postelmans L, Pasteels B, et al. Genetic biomarkers in the VEGF pathway predicting response to anti-VEGF therapy in age-related macular degeneration. *BMJ Open Ophthalmol*. 2019; 4(1): e000273. 10.1136/bmjophth-2019-000273 PMC6936450 31909188

[45] Fernández-Vega B, García M, Olivares L, et al. The association study of lipid metabolism gene polymorphisms with AMD identifies a protective role for APOE-E2 allele in the wet form in a Northern Spanish population. *Acta Ophthalmol*. 2020; 98(3): e282–e291. 31654486 10.1111/aos.14280

[46] McKay GJ, Patterson CC, Chakravarthy U, et al. Evidence of association of APOE with age-related macular degeneration: a pooled analysis of 15 studies. *Hum Mutat*. 2011; 32: 1407–1416. 21882290 10.1002/humu.21577 PMC3217135

[47] Bakbak B, Ozturk BT, Zamani AG, et al. Association of apolipoprotein E polymorphism with intravitreal ranibizumab treatment outcomes in age-related macular degeneration. *Curr Eye Res*. 2016; 41: 862–866. 26398858 10.3109/02713683.2015.1067325

[48] Wickremasinghe SS, Xie J, Lim J, et al. Variants in the APOE gene are associated with improved outcome after anti-VEGF treatment for neovascular AMD. *Invest Ophthalmol Vis Sci*. 2011; 52(7): 4072–4079. 21245410 10.1167/iovs.10-6550 PMC3175959

[49] Kim IK, Ji F, Morrison MA, et al. Comprehensive analysis of CRP, CFH Y402H and environmental risk factors on risk of neovascular age-related macular degeneration. *Mol Vis*. 2008; 14: 1487–1495. 18704199 PMC2515825

[50] Feng X, Xiao J, Longville B, et al. Complement factor H Y402H and C-reactive protein polymorphism and photodynamic therapy response in age-related macular degeneration. *Ophthalmology*. 2009; 116: 1908–1912. 19692124 10.1016/j.ophtha.2009.03.011

[51] Tosi GM, Caldi E, Neri G, et al. HTRA1 and TGF-β1 concentrations in the aqueous humor of patients with neovascular age-related macular degeneration. *Invest Ophthalmol Vis Sci*. 2017; 58: 162–167. 28114575 10.1167/iovs.16-20922

[52] Cruz-Gonzalez F, Cabrillo-Estevez L, Rivero-Gutierrez V, Sanchez-Jara A, De Juan-Marcos L, Gonzalez-Sarmiento R. Influence of CFH, HTRA1 and ARMS2 polymorphisms in the response to intravitreal ranibizumab treatment for wet age-related macular degeneration in a Spanish population. *Int J Ophthalmol*. 2016; 9: 1304–1309. 27672596 10.18240/ijo.2016.09.12 PMC5028666

[53] Kang HK, Yoon MH, Lee DH, Chin HS. Pharmacogenetic influence of LOC387715/HTRA1 on the efficacy of bevacizumab treatment for age-related macular degeneration in a Korean population. *Korean J Ophthalmol*. 2012; 26: 414–422. 23204795 10.3341/kjo.2012.26.6.414 PMC3506814

[54] McKibbin M, Ali M, Bansal S, et al. CFH, VEGF and HTRA1 promoter genotype may influence the response to intravitreal ranibizumab therapy for neovascular age-related macular degeneration. *Br J Ophthalmol*. 2012; 96: 208–212. 21558292 10.1136/bjo.2010.193680

[55] Park UC, Shin JY, McCarthy LC, et al. Pharmacogenetic associations with long-term response to anti-vascular endothelial growth factor treatment in neovascular AMD patients. *Mol Vis*. 2014; 20: 1680–1694. 25558172 PMC4278401

[56] Bobadilla M, Pariente A, Oca AI, Peláez R, Pérez-Sala Á, Larráyoz IM. Biomarkers as predictive factors of anti-VEGF response. *Biomedicines*. 2022; 10: 1003. 35625740 10.3390/biomedicines10051003 PMC9139112

[57] Oca AI, Pérez-Sala Á, Pariente A, et al. Predictive biomarkers of age-related macular degeneration response to anti-VEGF treatment. *J Pers Med*. 2021; 11: 1329. 34945801 10.3390/jpm11121329 PMC8706948

[58] Wei X, Ting DSW, Ng WY, Khandelwal N, Agrawal R, Cheung CMG. Choroidal Vascularity Index: a novel optical coherence tomography based parameter in patients with exudative age-related macular degeneration. *Retina*. 2017; 37: 1120–1125. 27632714 10.1097/IAE.0000000000001312

[59] Borrelli E, Sacconi R, Klose G, de Sisternes L, Bandello F, Querques G. Rotational three-dimensional OCTA: a notable new imaging tool to characterize type 3 macular neovascularization. *Sci Rep*. 2019; 9: 17053. 31745216 10.1038/s41598-019-53307-x PMC6863896

