# Supplementary material for: Influence of Genetic Polymorphisms on the Short-Term Response to Ranibizumab in Patients With Neovascular Age-Related Macular Degeneration
Source: Invest Ophthalmol Vis Sci. 2023 Oct 20;64(13):34. doi: 10.1167/iovs.64.13.34 (PMC10599160; doi:10.1167/iovs.64.13.34)
Supplement: Supplement 1 [file iovs-64-13-34_s001.pdf]

Supplementary Material: Predictive value of SLC16A8, HTRA1, CRP and CYP2J polymorphisms in the short-term response to naïve intravitreal ranibizumab treatment for neovascular Age related Macular Degeneration.

Laura García-Quintanilla[1,2,3†], Pablo Almuiña Varela[2,4†], Olalla Maroñas[5,6,7,8], Almudena Gil-Rodriguez[5,6], María José Rodríguez-Cid[4,] María Gil Martinez[4], Maximino J Abraldes[4,9,10], Francisco Gomez-Ulla[9] , Miguel González-Barcia[1,2], Cristina Mondelo-Garcia[1,2], Raquel Cruz[6,7], Ana Estany-Gestal[11], Maribel Fernández-Rodríguez [4,9,10]*, Anxo Fernández-Ferreiro[1,2]*

Table 1. Distribution of polymorphisms in our population.

Gene	SNP	Allele	Minor allele	MAF	HWE	Missing
**Immune response and inflammation**						
CRP	rs1205	C/T	T	37,2	0,5200	0
	rs1572970	A/G	G	31,4	0,4957	0
	rs1800947	C/G	G	5,8	1,0000	0
	rs1417938	T/A	A	25,6	0,7008	0
	rs876537	C/T	T	43,0	0,5403	0
	rs2808635	T/G	G	27,9	0,4586	0
	rs3093077	A/C	C	8,1	1,0000	0
C3	rs2230205	C/T	T	12,8	1,0000	0
	rs1389623	G/A	A	10,5	1,0000	0
	rs1047286	G/A	A	11,6	0,4455	0
	rs2230199	G/C	C	14,0	1,0000	0
	rs2250656	T/C	C	31,4	0,1673	0
CFH	rs1061170	C/T	T	43,0	0,3507	0
	rs1065489	G/T	T	17,4	0,5874	0
	rs10737680	A/C	C	22,1	0,0931	0
	rs10922109	C/A	A	22,1	0,0931	0
	rs3766404	T/C	C	9,3	1,0000	0
	rs380390	G/C	C	39,5	0,1189	0
	rs551397	C/T	T	14,0	0,5763	0
	rs570618	T/G	G	43,0	0,3507	0
	rs800292	G/A	A	14,0	0,5763	0
	rs800292	G/A	A	14,0	0,5763	0
CFB;NELFE;SKIV2L; MIR1236;C2	rs2072633	G/A	A	34,9	0,3107	0
CFB;NELFE;C2-AS1;MIR1236;C2	rs1048709	G/A	A	17,4	0,5874	0
CFB;NELFE;C2-AS1;C2	rs4151667	T/A	A	4,7	1,0000	0
	rs547154	G/T	T	2,3	1,0000	0
CFB;C2-AS1;C2	rs9332739	G/C	C	4,7	1,0000	0
NELFE;DXO;SKIV2L; MIR1236;STK19	rs429608	G/A	A	16,3	0,0595	0

IL1B	rs1143627	A/G	G	27,9	0,7044	0
IL1RL1	rs1041973	C/A	A	32,6	0,4855	0
IL1RAP	rs4624606	T/A	A	27,9	0,7044	0
CXCL8	rs4073	T/A	A	38,4	1,0000	0
	rs2227306	C/T	T	34,9	1,0000	0
TCF4	rs111947783	G	G	0,0	NA	0
TNF;LTB;LTA; LOC100287329	rs1800629	G/A	A	5,8	1,0000	0
	rs361525	G/A	A	7,0	0,1701	0
TNFRSF10A;LOC389641	rs13278062	T/G	G	46,5	0,3580	0
STAT3	rs744166	A/G	G	44,2	1,0000	0
**Angiogenesis**						
VEGFA	rs3025000	C/T	T	22,1	1,0000	0
	rs1413711	T/C	C	38,4	0,5252	0
	rs699947	A/C	C	39,5	0,3524	0
	rs833061	C/T	T	39,5	0,3524	0
VEGFC	rs12054587	G/A	A	11,6	0,4455	0
	rs3775194	G/C	C	33,7	0,7362	0
KDR	rs7691507	T/C	C	24,4	0,6879	0
	rs4576072	T/C	C	18,6	0,6148	0
	rs6828477	T/C	C	41,9	0,2075	0
NRP1	rs2229935	G/A	A	22,1	1,0000	0
	rs2247383	G/A	A	37,2	0,5200	0
	rs2804495	T/G	G	20,9	0,1657	0
SERPINF2;SERPINF1	rs12603486	G/A	A	34,9	0,7362	0
PGF	rs2268614	C/T	T	33,7	1,0000	0
FGFR2	rs2981582	A/G	G	45,3	0,7610	0
FLT1	rs7993418	A/G	G	25,6	0,2396	0
	rs9513070	A/G	G	39,5	0,7573	0
	rs9582036	A/C	C	36,0	0,1815	0
FLT4	rs55667289	G	G	0,0	NA	0
SYN3	rs5749482	G/C	C	4,7	1,0000	0
	rs5754227	T/C	C	4,7	1,0000	0
**Extracellular matrix and cellular adhesion**						
COL8A1	rs13081855	G/T	T	8,1	0,2324	0
	rs13095226	T/C	C	9,3	0,0283	0
PTGFR	rs3753380	C/T	T	17,4	0,5874	0
	rs3766355	C/A	A	16,3	0,5708	0
PLA2G12A	rs2285714	C/T	T	37,2	0,0201	0
TGFBR1	rs334353	T/G	G	26,7	1,0000	0
PLEKHA1	rs4146894	T/C	C	39,5	1,0000	0
ABCA4	rs1800553	C	C	0,0	NA	0
	rs1800555	C	C	0,0	NA	0
**Lipid metabolism and transport**						
APOC1;APOC1P1	rs4420638	A/G	G	8,1	1,0000	0

APOC1;TOMM40;APOE	rs405509	T/G	G	44,2	0,7594	0
	rs429358	T/C	C	3,5	1,0000	0
	rs7412	C/T	T	4,7	1,0000	0
	rs769449	G/A	A	3,5	1,0000	0
CETP	rs3764261	C/A	A	30,2	0,4735	0
	rs708272	G/A	A	43,0	0,0647	0
NLRC5;CETP	rs5882	A/G	G	33,7	0,7362	0
CYP2J2	rs890293	C/A	A	4,7	1,0000	0
CYP4F2	rs1558139	G/A	A	41,9	1,0000	0
**Cell survival and stress response**						
HTRA1;ARMS2	rs10490923	G/A	A	7,0	1,0000	0
	rs10490924	G/T	T	46,5	0,7596	0
	rs11200638	G/A	A	45,0	0,2184	7
	rs2736911	C/T	T	17,4	0,0912	0
	rs3750846	T/C	C	46,5	0,7596	0
HIF1A;HIF1A-AS1	rs10146037	T/C	C	10,5	0,3721	0
EPAS1	rs9679290	G/C	C	45,3	1,0000	0
SIRT1	rs12778366	T/C	C	10,5	0,3721	0
RAD51B	rs8017304	A/G	G	47,7	0,7643	0
	rs2588809	C/T	T	18,6	1,0000	0
**Other**						
LOC101928635	rs493258	C/T	T	44,0	1,0000	2,3
	rs10468017	C/T	T	22,1	0,6563	0
	rs920915	C/G	G	46,5	1,0000	0
LOC100506368;<br>FZD4;PRSS23	rs10898563	A/G	G	29,1	0,0737	0
LOC285857	rs4711751	T/C	C	40,7	0,3402	0
AGER	rs1800625	A/G	G	14,0	1,0000	0
	rs1800624	A/T	T	23,3	0,6666	0
LINC00243	rs3130783	A/G	G	25,6	0,2396	0
ERCC6	rs3793784	G/C	C	37,2	0,1972	0
	rs6987702	T/C	C	21,4	0,6558	2,3
SLC16A8;PICK1;BAIAP2L2	rs8135665	C/T	T	27,9	1,0000	0
B3GLCT	rs9542236	T/C	C	41,9	0,3596	0
	rs12678919	A/G	G	11,6	1,0000	0
	rs142450006	TTTC/-	-	10,5	1,0000	0
	rs201559	T/C	C	43,0	0,7562	0
	rs4351376	G	G	0,0	NA	0
MCUB	rs4698775	T/G	G	33,7	1,0000	0
VWA3A	rs55732851	G/A	A	10,5	0,3721	0
KCTD10;UBE3B	rs56209061	G/A	A	9,3	1,0000	0
FAAP100;NPLOC4	rs6565597	C/T	T	38,4	1,0000	0
ADAMTS9-AS2	rs6795735	C/T	T	46,5	1,0000	0

MAF: Minor allele frequency, HWE: Hardy–Weinberg equilibrium, SNP: Single-nucleotide polymorphism

Table 2. Results of the association with response for each SNP under different genetic models.

Gen	SNP	Co-dominant	dominant	recessive	log-additive
**Immune response and inflammation**					
CRP	rs1205	0,1477	0,2022	**0,0660**	0,0717
	rs1572970	0,7179	0,8068	**0,4164**	0,5908
	rs1800947	**0,2285**	NA	NA	NA
	rs876537	0,3054	**0,1262**	0,4565	0,1573
	rs1417938	0,7738	**0,4739**	0,8658	0,5025
	rs2808635	0,8736	**0,6578**	0,8658	0,7497
	rs3093077	**0,0325**	NA	NA	NA
C3	rs2230205	**0,4237**	NA	NA	NA
	rs1389623	**0,4565**	NA	NA	NA
	rs1047286	0,8377	**0,4565**	1,0000	0,8377
	rs2230199	1,0000	**0,5425**	1,0000	1,0000
	rs2250656	0,4056	**0,2022**	0,8658	0,2967
CFH	rs1061170	0,4733	**0,3685**	0,5596	0,7516
	rs1065489	0,9786	0,8643	0,8658	**0,8402**
	rs10737680	**0,7085**	NA	NA	NA
	rs10922109	**0,7085**	NA	NA	NA
	rs3766404	**0,7143**	NA	NA	NA
	rs380390	0,8738	0,6176	0,8064	**0,6126**
	rs551397	**0,3685**	NA	NA	NA
	rs570618	0,4733	**0,3685**	0,5596	0,7516
	rs800292	**0,3685**	NA	NA	NA
CFB;NELFE;C2AS1;MIR1236;C2	rs1048709	0,1495	**0,0604**	0,4950	0,1495
CFB;NELFE;SKIV2L;MIR1236;C2	rs2072633	0,1845	0,4739	**0,0660**	0,1671
CFB;NELFE; C2-AS1;C2	rs4151667	**0,1888**	NA	NA	NA
	rs547154	**0,8658**	NA	NA	NA
CFB;C2-AS1;C2	rs9332739	**0,1888**	NA	NA	NA
NELFE;DXO;SKIV2L; MIR1236;STK19	rs429608	0,4980	**0,4237**	0,6912	0,6774
IL1B	rs1143627	0,4000	**0,1819**	0,8064	0,2629
IL1RAP	rs4624606	**0,1621**	0,1819	0,4045	0,5151
IL1RL1	rs1041973	0,9223	0,9769	**0,6912**	0,8503
CXCL8	rs4073	0,3048	0,5549	**0,1249**	0,2374
	rs2227306	0,4618	0,5149	**0,2285**	0,2946
TCF4	rs111947783	NA	NA	NA	NA
TNF;LTB;LTA;LOC100287329	rs1800629	**0,8406**	NA	NA	NA
	rs361525	0,7843	**0,2285**	1,0000	0,7843
TNFRSF10A;LOC389641	rs13278062	0,3190	0,2350	0,1827	**0,1338**
STAT3	rs744166	0,8933	0,8643	**0,7143**	0,9257
**Angiogenesis**					
VEGFA	rs3025000	0,5916	0,7477	**0,4950**	0,5916
	rs1413711	0,4831	0,8107	**0,2285**	0,4471

	rs699947	0,4696	0,5943	**0,2285**	0,3220
	rs833061	0,4696	0,5943	**0,2285**	0,3220
VEGFC	rs12054587	0,8377	**0,4565**	1,0000	0,8377
	rs3775194	0,6928	0,9769	**0,4045**	0,6942
KDR	rs7691507	0,4733	**0,2213**	0,6912	0,2558
	rs4576072	0,6500	0,9029	**0,4950**	0,6500
	rs6828477	0,7515	0,8358	**0,4503**	0,5800
NRP1	rs2229935	0,9161	0,7477	0,8658	**0,8348**
	rs2247383	0,4495	0,2213	0,4521	**0,2200**
	rs2804495	**0,9769**	NA	NA	NA
SERPINF2;SERPINF1	rs12603486	0,4798	0,8068	**0,2319**	0,4441
PGF	rs2268614	0,4843	0,7085	**0,2285**	0,4049
FGFR2	rs2981582	0,5155	**0,3685**	0,6713	0,7272
FLT1	rs7993418	0,4324	**0,2894**	0,4419	0,4324
	rs9513070	0,0926	0,2907	**0,0635**	0,0637
	rs9582036	0,6601	0,8107	**0,4164**	0,8736
FLT4	rs55667289	NA	NA	NA	NA
SYN3/TIMP3	rs5749482	**0,4045**	NA	NA	NA
	rs5754227	**0,4045**	NA	NA	NA
**Extracellular matrix and cellular adhesion**					
COL8A1	rs13081855	0,4796	**0,2319**	0,4419	0,4796
	rs13095226	0,3469	0,2319	**0,1894**	0,3469
PTGFR	rs3753380	0,6420	0,6176	**0,4950**	0,6420
	rs3766355	**0,2350**	NA	NA	NA
PLA2G12A	rs2285714	0,9667	**0,8643**	0,8658	0,9359
TGFBR1	rs334353	0,5238	**0,2563**	0,6912	0,2815
PLEKHA1	rs4146894	0,2778	0,4953	**0,1111**	0,1901
ABCA4	rs1800553	NA	NA	NA	NA
	rs1800555	NA	NA	NA	NA
**Lipid metabolism and transport**					
APOC1;APOC1P1	rs4420638	**0,9383**	NA	NA	NA
APOC1;TOMM40; APOE	rs405509	0,1368	**0,0646**	0,9860	0,2321
	rs429358	**0,6912**	NA	NA	NA
	rs7412	**0,8064**	NA	NA	NA
	rs769449	**0,6912**	NA	NA	NA
CETP	rs3764261	0,7300	**0,4313**	0,8406	0,5036
	rs708272	0,8305	0,7592	**0,5425**	0,6017
NLRC5;CETP	rs5882	0,8347	**0,5521**	0,8064	0,5674
CYP2J2	rs890293	**0,0314**	NA	NA	NA
CYP4F2	rs1558139	0,7488	0,9029	**0,4521**	0,6213
**Cell survival and stress response**					
HTRA1;ARMS2	rs10490923	**0,7579**	NA	NA	NA
	rs10490924	0,3093	**0,1315**	0,7603	0,2566
	rs11200638	0,0887	**0,0404**	0,8532	0,1655

	rs2736911	0,5865	0,8358	**0,4164**	0,8505
	rs3750846	0,3093	**0,1315**	0,7603	0,2566
HIF1A;HIF1A-AS1	rs10146037	0,4194	0,6713	0,4419	**0,4194**
EPAS1	rs9679290	0,2888	0,4020	**0,1245**	0,1578
SIRT1	rs12778366	1,0000	**0,6713**	1,0000	1,0000
RAD51B	rs2588809	0,4210	**0,3772**	0,4419	0,4210
	rs8017304	0,7516	0,9218	**0,4565**	0,6140
**Oher**					
LOC101928635	rs493258	0,8222	**0,6991**	0,7324	0,9490
	rs10468017	1,0000	**0,9769**	1,0000	1,0000
	rs920915	0,9775	**0,8358**	0,9860	0,8857
LOC100506368; FZD4;PRSS23	rs10898563	0,7403	0,8068	**0,4419**	0,7403
LOC285857	rs4711751	0,9721	0,8643	0,8406	**0,8176**
AGER	rs1800625	1,0000	**0,5425**	1,0000	1,0000
	rs1800624	0,5615	0,3500	0,4164	**0,2864**
LINC00243	rs3130783	0,2902	**0,2894**	1,0000	0,2902
ERCC6	rs3793784	0,6877	**0,3869**	0,7143	0,4461
	rs6987702	0,5301	0,6624	**0,4524**	0,5301
SLC16A8;PICK1;BAIAP2L2	rs8135665	0,1242	**0,0420**	0,6912	0,0684
B3GLCT	rs9542236	0,7133	0,9646	**0,4413**	0,6523
	rs12678919	**0,2958**	NA	NA	NA
	rs142450006	**0,1245**	NA	NA	NA
	rs201559	0,6930	**0,4020**	0,9383	0,5340
	rs4351376	NA	NA	NA	NA
MCUB	rs4698775	0,4843	0,7085	**0,2285**	0,4049
VWA3A	rs55732851	0,8225	0,7143	**0,4419**	0,8225
KCTD10;UBE3B	rs56209061	0,2148	NA	NA	NA
FAAP100;NPLOC4	rs6565597	0,4608	0,9646	**0,2319**	0,5193
ADAMTS9-AS2	rs6795735	0,6415	**0,3685**	0,9860	0,5579

* The model selected based on significance is marked in bold.
